# Mycobacterium marinum Cutaneous Infection: A Series of Three Cases and Literature Review

**DOI:** 10.7759/cureus.31787

**Published:** 2022-11-22

**Authors:** Inês C Gonçalves, Isabel Furtado, Maria João Gonçalves, Sandra Xará

**Affiliations:** 1 Infectious Disease, Centro Hospitalar e Universitário do Porto, Porto, PRT; 2 Infectious Disease, Hospital Pedro Hispano, Matosinhos, PRT

**Keywords:** aquarium infection, skin lesions, dermatology, infectious diseases, mycobacterium marinum

## Abstract

*Mycobacterium marinum* is a non-tuberculous mycobacteria present in natural and non-chlorinated bodies of water. It is a known fish pathogen but can also cause human disease. It usually causes cutaneous lesions but in rare cases may originate more invasive diseases with the involvement of deep structures. We describe three cases of patients with cutaneous infection by *M. marinum* evaluated in a tertiary care center, two with confirmed infection and one with a presumptive diagnosis based on clinical and epidemiological features. A brief bibliographic review of *M. marinum* infections is then presented to support the theme. We aim to alert one to the difficulties in establishing the correct diagnosis of this infection, emphasize the importance of a high degree of suspicion for its identification, and review the therapeutic management options.

## Introduction

*Mycobacterium marinum* is a non-tuberculous mycobacterium (NTM) associated with aquatic environments that can cause human infection [1,2]. It is an endemic fish pathogen with worldwide distribution and it may be present in both fresh and salt water, especially in relatively still or stagnant water such as fish tanks or non-chlorinated swimming pools [3,4]. The prevalence of mycobacteria infection in ornamental fishes has been reported as high as 47% [1,2]. Human infection is acquired by direct inoculation through a breach in the skin in an infectious aquatic environment or from direct contact with contaminated fish or shellfish [5]. Fish enthusiasts and participants in waterborne activities are at greater risk of infection [5,6]. The incubation period is usually less than four weeks but it can be considerably longer with case reports of up to nine months [7]. From the NTM group, *M. marinum* is one of the leading causes of extra respiratory human disease worldwide, affecting both immunocompetent and immunocompromised hosts [3,8]. Human infection is characterized by cutaneous and/or subcutaneous involvement presenting as solitary or multiple nodular or ulcerated lesions, usually on the extremities of the upper limbs such as a finger or the hand [3]. Lymphangitic dissemination can also occur in a sporotrichosis pattern in up to one-third of cases [9,10]. In immunosuppressed hosts or when there is a delay in diagnosis, *M. marinum *infection can cause deep structure and invasive disease including tenosynovitis, septic arthritis, or osteomyelitis [3,4]. We present three cases of cutaneous infection by *M. marinum* evaluated and treated in a tertiary care hospital in Portugal. Two cases had *M. marinum* mycobacterial confirmation and one was a presumed diagnosis considering the clinical-pathologic data and epidemiological context.

## Case presentation

Case one

**Figure 1:**
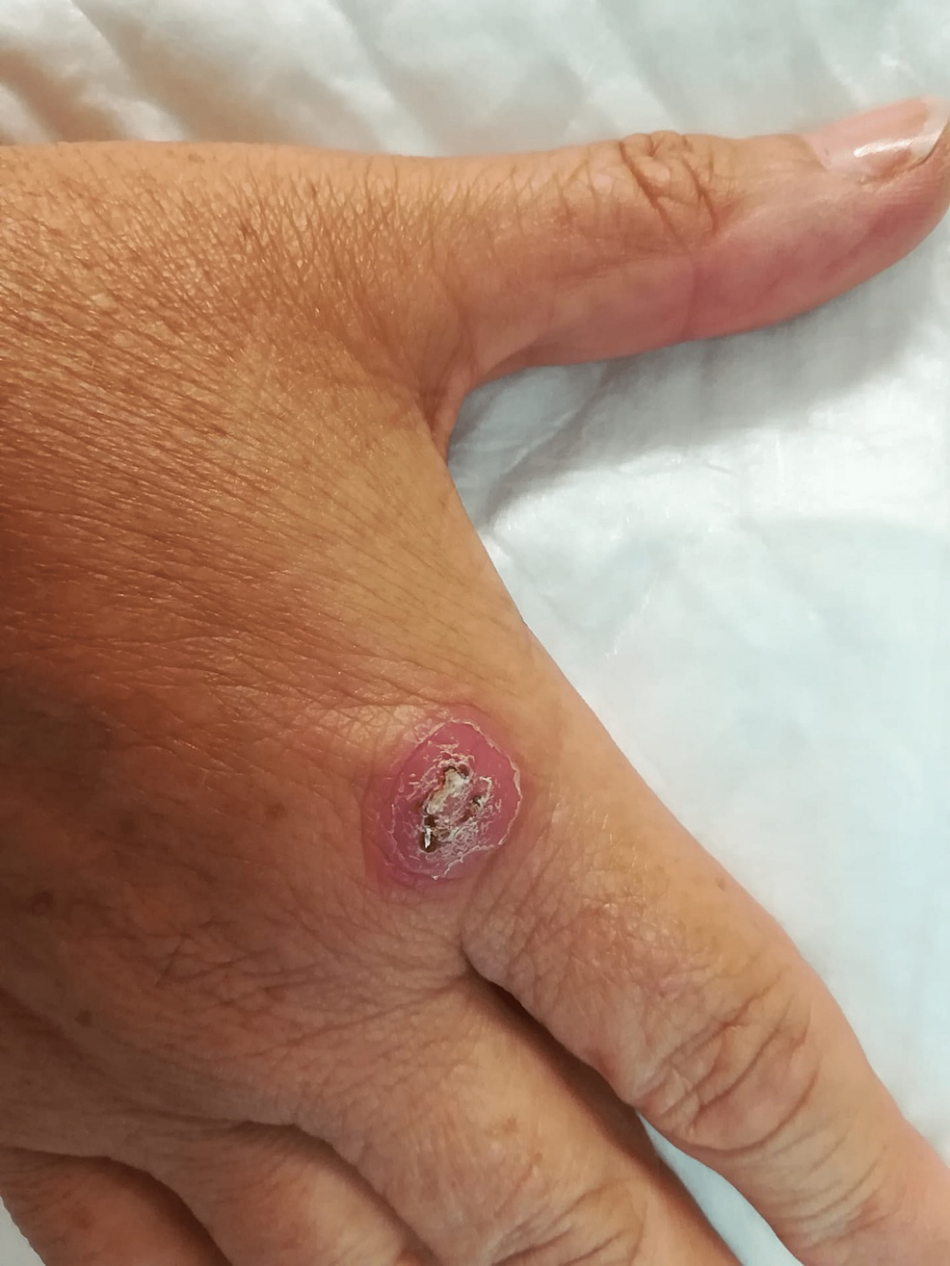
Erythematous papule with exulceration

A healthy 58-year-old woman presented with an ulcerated skin lesion on the dorsum of the right hand just above the metacarpophalangeal joint of the right index finger that had appeared 12 months before presentation and never seemed to heal. The patient reported some associated erythema but no pain, pruritus, exudate, or other local symptoms. During this period she never presented any systemic complaints such as fever, malaise, myalgias, weight loss, or hyperhidrosis. She was a kindergarten teacher and had frequent contact with a tropical fish aquarium in the classroom. The lesion was characterized as an erythematous papule with exulceration (Figure 1). A previous biopsy was inconclusive and no clinical response was observed with a seven-day course of amoxicillin/clavulanate (875/125mg every 12 hours, oral). A second biopsy was performed and histological analysis revealed granulomatous dermatitis. No acid-fast bacilli (AFB) were visualized on direct examination with Ziehl-Neelsen (ZN) staining. Mycobacteria grew on solid media cultures of the biopsied tissue, with ulterior identification by the polymerase chain reaction (PCR) technique of* M. marinum* or *M*ycobacterium *ulcerans *genetic material. There were no other microbiologic isolations in bacteriological studies. Although the interferon-gamma release assay (IGRA) was positive, the patient had had no previous tuberculosis diagnosis or known contact with infected individuals, and an active infection was excluded through symptom inquiry and chest radiograph. A diagnosis of cutaneous infection by *M. marinum* was assumed and combined treatment was started with ethambutol (20mg/kg/day, oral) and rifampin (600mg/day, oral). Gradual improvement occurred over two months and surgical resection of the remaining lesion was posteriorly performed. Another full month of combination therapy was administered afterward (total therapy duration of three months) with full resolution and no relapse observed in the 24-month follow-up period.

Case two

**Figure 2:**
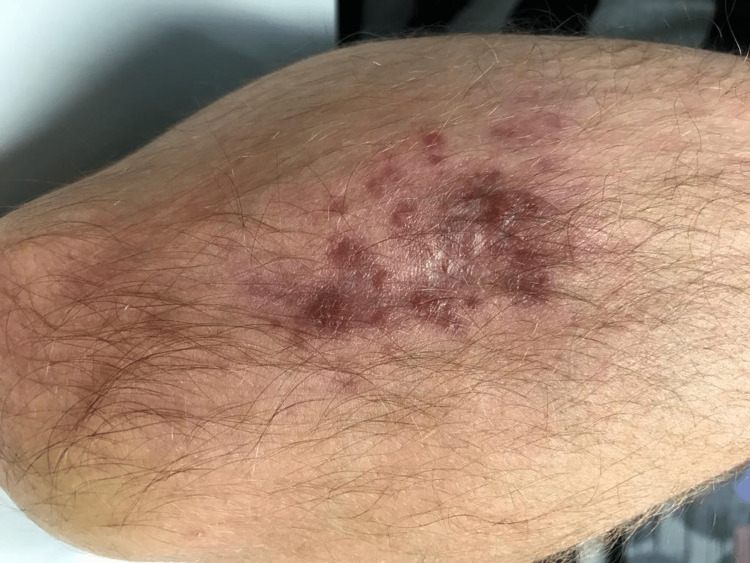
Various nodular lesions grouped in the forearm

A 38-year-old fish farmer with a history of lymphoma (diagnosed five years before presentation, treated, and considered in remission), presented with various nodular lesions grouped in the forearm covering an area of 5cm (Figure 2). The first lesion appeared three months before presentation, with no accompanying local symptoms such as pain, desquamation, exudate, or pruritus, and no systemic symptoms. A biopsy of this lesion revealed a granulomatous necrotizing dermatitis, with identification of AFB on direct examination. Mycobacteria grew in the culture of the biopsied tissue with ulterior identification by PCR technique of *M. marinum* or *M. ulcerans*. The IGRA test was positive although the patient had had no previous tuberculosis diagnosis or known contact with infected individuals and an active infection was excluded through symptom inquiry and chest radiograph. A diagnosis of cutaneous infection by *M. marinum* was assumed. Combination therapy with ethambutol (20mg/kg/day, oral) and rifampin (600mg/day, oral) was started. There was a progressive reduction in the number and size of lesions over 10 months but some lesions persisted which prompted surgical resection with a posterior skin graft. Two more months of antibiotic treatment were administered (a total treatment time of 12 months). There was complete resolution of the lesions and no documented relapse for a follow-up period of 12 months.

Case three

**Figure 3:**
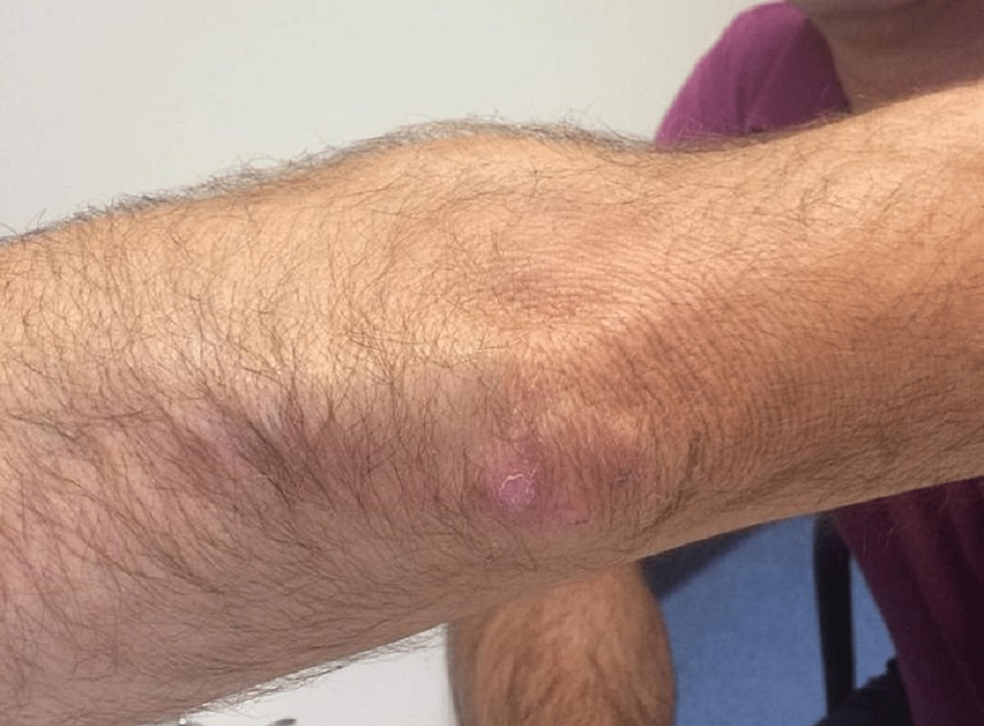
Ulcerated lesion located on the elbow

A 49-year-old fish farmer working in the same fishpond as the patient from case two, presented six months after his colleague with an ulcerated lesion located on the elbow (Figure 3). It had appeared in the month previous to the presentation with accompanying pruritus but no other local or systemic symptoms. A biopsy of the lesion revealed granulomatous dermatitis with the identification of AFB on direct examination (ZN staining). The bacteriological and mycobacterial cultures and molecular tests were all negative. The IGRA test was negative. Considering the epidemiological context (occupational exposure risk and confirmed infection in a work colleague) and the presence of AFB on tissue specimens, a cutaneous infection by *M. marinum* was presumed. The pinch biopsy almost wholly removed the lesion and treatment was completed with a posterior course of antibiotic therapy with ethambutol (20mg/kg/day, oral) and clarithromycin (500mg/12 hours, oral) for two months, with optimal clinical response and resolution and no relapse in the follow-up period of eight months. The choice of clarithromycin instead of rifampin was due to the interaction of rifampin with itraconazole that the patient was taking to treat onychomycosis. In both cases two and three, public health authorities were involved. Adequate aquarium cleaning was recommended. No more secondary cases are known.

## Discussion

*Mycobacterium marinum* cutaneous infection is relatively rare and its correct diagnosis can be difficult given its insidious and nonspecific presentation. Frequently, there is a considerable time delay between the symptoms' appearance and the identification of the etiologic agent [4]. As described in the three reported clinical cases, this kind of infection must be considered when key diagnostic elements are present. A high index of suspicion is sustained by the combination of some factors such as a cutaneous lesion with poor response to conventional antibiotic treatments and a history of aquatic exposure (mainly fish farmers and aquarium enthusiasts) [8-10].

Cutaneous lesions caused by *M. marinum* are non-pathognomic and thus the combination of both histological analysis of the affected tissue and mycobacterial studies (cultures and molecular tests) are necessary for its correct identification. There are reports of positive cultures in 70% to 80% of cases [1], but this number could be increased if proper attention is paid to specimen collection and correct incubation temperature [3,4]. The optimal growing temperature of *M. marirum* in cultures is around 30°C (lower than for usual pathogens), whereas small colonies or no growth is observed at 37°C [3,4]. This could explain the typical localization of the lesions: upper or lower extremities and sometimes in the tip of the nose [9]. In the three presented cases, the lesions were found solely in the upper extremities as this was the only body part in contact with contaminated water. In both cases two and three, protective gloves covering hands and wrists were used when contacting with the fishponds, rendering a more proximal involvement of the arms than in case one.

Isolation of *M. marinum* is always of clinical significance (despite the number of colonies) since it usually does not grow in the laboratory environment or from the uninfected human body [3]. Currently, commercially available PCR techniques are unable to distinguish between *M. marinum* and *M.ulcerans* as these two mycobacteria share 98% of their genome [3]. Although IGRA tests were not found to be useful as a diagnostic method for *M. marinum* infection [3], they may be positive in patients with this infection as *M. marinum* shares with *Mycobacterium tuberculosis* the specific antigens early secreted antigenic target (ESAT)-6 and culture filtrate protein (CFP)-10 that may elicit a positive result in such a test [3]. 

In the first two cases, the diagnosis was sustained by mycobacterial growth in culture and a positive *M. marinum*/*M. ulcerans* PCR test as well as a positive IGRA test. In the third case, although negative on microbiologic tests, the epidemiological context was decisive for the diagnosis: aquatic environment occupational exposure and a work colleague with confirmed *M. marinum *infection.

The differential diagnosis for *M*. *marinum* infection includes other NTM known to cause cutaneous infection (*Mycobacterium chelonae, M. ulcerans, Mycobacterium * *haemophilum, Mycobacterium* ​​​​*fortuitum, and M. tuberculosis*), sporotrichosis, and noninfectious diseases such as sarcoidosis, skin tumors, and foreign-body reactions [3].

Regarding treatment, as there are no comparative trials of different treatment regimens and there is no consensus for an adequate duration of therapy, the statement endorsed by the Infectious Diseases Society of America/American Thoracic Society (IDSA/ATS) regarding diagnosis, treatment, and prevention of nontuberculous mycobacterial diseases from 2007 [4] is useful for guiding treatment choices. Although spontaneous remission without treatment has been reported in immunocompetent individuals with small lesions, usually combination antibiotic treatment is needed for complete resolution of lesions [8,10]. A reasonable approach is to treat with two active agents until the lesions heal and then for one or two additional months, typically for a three to four-month total period of treatment guided mainly by clinical response and considering both the extension and severity of the infection and the presence of underlying disorders [3,4,8]. By standard susceptibility testing, *M. marinum* organisms are susceptible to rifampin, rifabutin, and ethambutol and resistant to isoniazid and pyrazinamide. Isolates are usually also susceptible to clarithromycin and sulfonamides and susceptible or intermediately susceptible to tetracyclines [4,8]. The most frequent agents used are clarithromycin, rifampin, and ethambutol with the choice of the combination regimen being mostly based on the physician’s experience and guided by the patient’s tolerability, as in the described cases. Surgical excision is not universally recommended but may be indicated especially for diseases involving the closed spaces of the hand, extensive lesions, and antibiotic refractory lesions [3,4]. In two of the described cases, surgical excision was needed in association with antibiotics. Susceptibility testing is not routinely recommended and should be reserved for cases of treatment failure. In the presented cases the choice of treatment regimens was rifampin in cases one and two, and clarithromycin in case three, all combined with ethambutol. Case two was particularly challenging given the extent of the lesions, highlighting the role that surgical excision can play in treating this infection. The prognosis of *M. marinum* cutaneous infection is usually favorable if it is appropriately treated before deep soft tissue and/or bone involvement [4,8,9]. The high clinical suspicion favored adequate and prompt treatment in the presented cases, resulting in an excellent prognosis in all cases.

## Conclusions

Activities that bring people into contact with fish tanks are increasing in popularity and seem to represent the main risk factors for *M. marinum* infections. The high prevalence of mycobacteria infection in ornamental fishes illustrates the importance of the use of adequate individual protective measures such as gloves for fish farmers or aquarium keepers. Establishing a diagnosis of *M. marinum* infection is not always straightforward given its insidious and nonspecific presentation, long incubation period, and the technical difficulties raised during the microbiological confirmation of the organism. In practice, diagnosis remains largely presumptive based on clinical-histological features and the response to treatment. The clinician should suspect *M. marinum* infection in the presence of poorly healing wounds, mainly in the upper extremities, and in persons with a history of exposure to aquariums. This three-case report aims to alert clinicians to this infection and difficulties in its diagnostic and therapeutic management, and emphasize the importance of the use of protective measures in individuals at risk.

## References

[1] Hashish E, Merwad A, Elgaml S (2018). Mycobacterium marinum infection in fish and man: epidemiology, pathophysiology and management; a review. Vet Q.

[2] Puk K, Guz L (2020). Occurrence of Mycobacterium spp. in ornamental fish. Ann Agric Environ Med.

[3] Aubry A, Mougari F, Reibel F, Cambau E (2017). Mycobacterium marinum. Microbiol Spectr.

[4] Griffith DE, Aksamit T, Brown-Elliott BA (2007). An official ATS/IDSA statement: diagnosis, treatment, and prevention of nontuberculous mycobacterial diseases. Am J Respir Crit Care Med.

[5] Johnson MG, Stout JE (2015). Twenty-eight cases of Mycobacterium marinum infection: retrospective case series and literature review. Infection.

[6] Lewis FM, Marsh BJ, von Reyn CF (2003). Fish tank exposure and cutaneous infections due to Mycobacterium marinum: tuberculin skin testing, treatment, and prevention. Clin Infect Dis.

[7] Jernigan JA, Farr BM (2000). Incubation period and sources of exposure for cutaneous Mycobacterium marinum infection: case report and review of the literature. Clin Infect Dis.

[8] Rallis E, Koumantaki-Mathioudaki E (2007). Treatment of Mycobacterium marinum cutaneous infections. Expert Opin Pharmacother.

[9] Franco-Paredes C, Marcos LA, Henao-Martínez AF, Rodríguez-Morales AJ, Villamil-Gómez WE, Gotuzzo E, Bonifaz A (2018). Cutaneous mycobacterial Infections. Clin Microbiol Rev.

[10] Bouceiro-Mendes R, Ortins-Pina A, Fraga A (2019). Mycobacterium marinum lymphocutaneous infection. Dermatol Online J.

